# Dialysis Access Maintenance: Plain Balloon Angioplasty

**DOI:** 10.1007/s00270-023-03441-x

**Published:** 2023-05-08

**Authors:** Lakshmi Ratnam, Narayan Karunanithy, Leto Mailli, Athanasios Diamantopoulos, Robert A. Morgan

**Affiliations:** 1 Department of Interventional Radiology, St George’s University Hospitals NHS Foundation Trust, London, UK; 2 Molecular and Clinical Sciences Research Institute, St George’s, University of London, London, UK; 3 Department of Interventional Radiology, Guys and St. Thomas NHS Foundation Trust, London, UK; 4 School of Biomedical Engineering & Imaging Sciences, Faculty of Life Sciences & Medicine, Kings College London, London, UK

**Keywords:** Haemodialysis, Access maintenance, Arteriovenous fistula, Balloon dilatation, Angioplasty, Percutaneous transluminal angioplasty

## Abstract

Plain balloon angioplasty remains the first-line treatment for dialysis access stenosis. This chapter reviews the outcomes of plain balloon angioplasty from cohort studies and comparative studies. Angioplasty outcomes are more favourable in arteriovenous fistulae (AVF) compared to arteriovenous grafts (AVG) with primary patency at 6 months ranging from 42–63% compared to 27–61%, respectively, and improved for forearm fistulae compared with upper arm fistulae. Higher pressures are required to treat stenoses in AVFs compared to AVGs. Outcomes are worse in more severe stenoses, increased patient age, previous interventions and fistulae that develop early stenoses. Major complication rates following angioplasty in dialysis access are between 3 and 5%. Repeat treatments and the use of adjuncts such as drug-coated balloons and stents can prolong the patency of dialysis access.

**Level of Evidence** No level of evidence (Review paper).

## Introduction

Subcutaneous arteriovenous fistulae were developed by Brescio and Cimino in 1966, providing vascular access for haemodialysis. This was followed by the introduction of expanded polytetrafluorethylene (ePTFE) grafts into clinical practice in 1976. These are the most established means of long-term haemodialysis. However, preserving the patency of these accesses remains challenging [1]. Stenosis can develop in the access circuit due to multiple factors and if untreated, can lead to reduced effectiveness of dialysis, progressive loss of function and thrombosis of the access circuit [2]. These outcomes all lead to significant morbidity and result in substantial economic cost [3, 4].

Plain balloon angioplasty (PBA), typically with high-pressure balloons, is considered to be the mainstay of treatment for dialysis access circuit stenoses and is indicated when there is an angiographically significant stenosis associated with clinical dysfunction [5]. Clinical manifestation of access dysfunction manifest broadly as disorders of inflow (needling difficulty, inability to achieve adequate dialysis flow speed, and poor fistula maturation) or outflow (arm swelling and prolonged bleeding) [5]. Although the outcomes of surgical revision have been shown to be comparable to angioplasty, the use of angioplasty can prolong the life of a fistula whilst preserving the option of surgical revision. Angioplasty also has the added advantage of allowing treatment of synchronous lesions [6–9]. In keeping with the latest KDOQI guidelines, the use of PBA must be factored in as part of the overall, individual patient’s pathway and long-term care of each patient (the ESKD (End Stage Kidney Disease) Life Plan) [5]. The aim of this article is to summarise the evidence for the use of PBA in maintaining functional access circuits.

## Materials and Methods

A narrative review of the literature was performed using Medline and Embase via Ovid to include studies published from 1980 to 2022. These were supplemented with citation searches from identified studies. Search terms used were haemodialysis, dialysis, access maintenance, arteriovenous fistula, balloon dilation, angioplasty, and percutaneous transluminal angioplasty. Cohort studies describing outcomes of PBA, comparative studies with more than 30 patients treated with drug-coated balloons (DCB) versus PBA with up to 1 year outcomes data, and stent graft versus PBA were included for analysis to review the outcomes of PBA. Studies of central venous stenosis, thrombosed access circuits, immature fistulae, and studies where more than 20% of patients underwent adjuvant stent procedures were excluded. Studies with mixed data were excluded if sufficient detailed breakdown was not provided to enable extraction of the relevant patient cohort (number of AVF versus AVG, number of patients receiving stents, location of access, patency rates at 6 and 12 months) (Table 1).

**Table 1:** Summary of search strategy

Items	Specification
Date of search	22/07/2022
Databases and sources searched	Medline, Embase via Ovid
Search terms	haemodialysis, dialysis, access maintenance, arteriovenous fistula, balloon dilatation, angioplasty, and percutaneous transluminal angioplasty
Timeframe	1980 to 2022
Exclusion criteria	Studies with central venous stenosis, thrombosed circuits, immature fistula, > 20% patients having adjuvant stent procedures, and mixed data studies with insufficient detail to extract relevant patient cohort for this chapter
Selection process	LR, LM

## Definitions

### Angiographic Stenosis

50% luminal narrowing compared with the normal vascular segment located adjacent to the stenosis [5].

### Elastic Recoil

Rebound of the vessel wall after undergoing PTA that results in recurrent narrowing.

### Resistant Stenotic Lesions

Lesions where < 30% residual stenosis following dilation with a standard high-pressure balloon is not achieved.

### Primary Patency

Interval following intervention that the lesion requires reintervention.

### Secondary Patency

Patency until access is surgically de-clotted, revised or abandoned. Multiple treatments including angioplasty and thrombectomy may be included in secondary patency [10].

### Technique for Balloon Angioplasty

Access is generally obtained from the venous side of the circuit as routine arterial puncture is documented to have higher potential for complications as well as longer monitoring required after the procedure [11, 12]. The lesion is crossed using standard wire and catheter techniques under fluoroscopic guidance. The balloon size is matched to the reference vessel diameter in an approximate 1:1 sizing [13]. In practice, balloon diameters of between 7 and 10 mm are usually utilised in the venous limb of AVFs (arteriovenous fistulas). Balloons that are 1 mm larger in diameter to the graft are usually used for treating AVGs (arteriovenous grafts). Smaller diameter balloons are used to treat arterial anastomotic stenoses to avoid over dilation of the surgical anastomosis. Balloon length is chosen depending on the length of the lesion being treated, taking care not to dilate the normal vessel if possible. Adequate treatment necessitates the obliteration of the ‘waist’ of the stenosis and may require the use of high-pressure balloons. Standard high-pressure balloons are rated to burst pressures of 20 atm with newer ultra-high-pressure balloons capable of producing up to 40 atm of pressure [14]. Various studies assessing the pressures required to overcome stenoses in AV fistula have found that higher pressures are required more often in AVF compared to AVGs. The mean pressure required to successfully obliterate the waist of the balloon in any stenosis was found to be between 15–17 atm [14, 15]. Published inflation times vary, but in general, most operators utilise inflation times of between 60–120 s. A few studies have attempted to study the effect of inflation times on the outcome and longer-term patency with inconclusive results [16, 17]. In the absence of evidence for the length of inflation time, most operators will perform a prolonged inflation and increase the balloon size in situations where recoil of the dilated stenosis is seen, with consideration of using stents or stent grafts if the problem is persistent. In cases where the stenosis is resistant, an increase in balloon pressure is required. A technically successful angioplasty is defined as achievement of less than 30% residual stenosis. In cases of multiple stenoses, all lesions that may contribute to the clinical dysfunction are treated [10]. Catheter-based flow measurements have been described as a problem-solving tool in patients with multiple stenoses to determine which lesions are in fact contributing to the clinical dysfunction [18]. Blood flow rates of less than 600 mL/min are taken as a measure of significant dysfunction [19]. Dilation of stenoses within fistulae can be very painful and the use of sedo-analgesia is advised for patient comfort whilst maintaining this as a day case procedure under local anaesthetic. In the authors institution, a combination of fentanyl and midazolam is utilised. Heparin is administered once a sheath is sited and in cases where the artery is accessed, glyceryl trinitrate is administered to avoid arterial spasm.

## Results

### Outcomes of PBA in Cohort Series

For a mixed population of patients, (both AVGs and AVFs), the primary patency rates post PTA for failing fistulas at 6 and 12 months range from 61–70% and 42–55%, respectively. For the same mixed group, the secondary patency rates at 6 and 12 months were 95 and 85%, respectively [8, 20]. The two groups were then assessed separately. The studies selected for reporting the outcome of PBA in AVF and AVG included in our analysis are summarised in Table 2.

**Table 2:** Outcomes of plain balloon angioplasty in cohort studies [1, 6, 8, 12, 13, 20–25]

Study, year (AVF/AVG)	Total cohort	Study design	Location of access	Primary Patency (%)		Secondary Patency (%)	
				6 mths	12 mths	6 mths	12 mths
**AVF**							
Glanz; 1984	51	Retrospective	NA	70	55	NA	NA
Turmel-Rodrigues; 2000	220	Retrospective (63) and Prospective (376)	RCF = 209 Upper arm = 74 (AVGs excluded)	67 57	51 35	NA NA	85 82
Clark; 2002	65	Retrospective	RCF = 37 BCF/BBF = 28	55	26	82	82
Rajan; 2004	151	Retrospective	RCF = 94	75	62	88	86
			BCF = 57	51	39	89	85
Heye; 2011	167	Retrospective	RCF = 70 Upper arm = 97	73	49	NA	84
Bountouris; 2014	159	Retrospective	RCF = 81 BCF = 56	61	42	89	85
Manou-Stathopoulou; 2019	124	Retrospective	RCF = 11 Upper arm = 113	68	56	83	77
**AVG**							
Beathard; 1992	536	Prospective	NA	61	38	NA	NA
Kanterman; 1995	93	Retrospective	Forearm loop graft	63	41	50	25
Safa; 1996	90	Prospective	Forearm = 83 Upper arm = 7	43	23	NA	82
Lumsden; 1997	40	Retrospective	Forearm = 11 Upper arm = 27 Femoral = 2	27	10	NA	NA

_Type of AVF (*RCF*—radiocephalic fistula, *BCF—*brachiocephalic fistula, *BBF*—brachiobasilic fistula) or AVG; *NA* not available_

For AVFs, the target lesion primary patency at 6 and 12 months range between 42–63% and 23–50.5%, respectively, based on analysis of the studies by Turmel-Rodrigues et al., Clark et al., Rajan et al. (2003 and 2004). AVFs primary patency rates obtained by averaging the breakdown data from Turmel-Rodrigues et al. and Rajan et al. (2004) [12, 13, 24, 26]. A more detailed analysis between the forearm and upper arm AVFs was conducted by Turmel-Rodrigues et al. who showed primary patency at 6 and 12 months to be superior for the forearm group (67% v 57% at 6 months and 51% v 35% at 12 months) [12]. No significant difference in secondary patency was demonstrated. Similarly, Rajan et al. (2004) demonstrated superior primary patency for radiocephalic versus upper arm fistulae (75% v 51% at 6 months, 62% versus 39% at 12 months) [24]. Once again, no significant difference was reported in secondary patency. These outcomes are supported by Heye et al. who also found improved technical success for PBA in radiocephalic compared to upper arm fistulae [21].

For AVGs, the target lesion primary patency at 6 and 12 months range 27–63% and 10–41%, respectively [1, 6, 22, 25].

Several studies have looked to identify patient and access circuit factors that may predict a favourable outcome for PBA. Neuen BL et al. found that fistulas developing early stenosis after fistula creation, longer lesion lengths and increased patient age (likely a reflection of quality of peripheral vasculature) were associated with primary patency loss after PBA [27]. Manou-Stathopoulou et al. identified patient age, non-white ethnicity, multiple previous interventions, thrombosis at the time of intervention and lesion length as predictors of poor outcome; and greater fistula age a predictor of good outcome [23]. Heye also identified greater fistula age as a positive predictor of success; and early stenoses, recurrence of stenosis and diabetes were predictors of poor outcome [21].

The major complication rates following PTA for failing AVFs ranged from 0 to 2.1% [12, 13, 21, 24], while for the AVGs ranged from 2.1 to 6% [1, 6, 22, 25]. Papers with mixed AVGs and AVFs reported complication rates of 3–5% [8, 20]. The most significant complications reported are thrombosis, rupture and dissection requiring either stent graft placement or surgical revision of the fistula.

### Outcomes of PBA in Comparative Series

#### Outcomes of PBA in AVF Drug-Coated Balloon Randomised Controlled Trials

There have now been several prospective studies comparing the effectiveness of PBA with drug-coated balloons (DCB). The studies that included 30 or more patients in the control PBA arm and reported outcomes for the PBA cohort up to 1 year are summarised in Table 3.

**Table 3:** Outcomes of plain balloon angioplasty in AVF DCB trials with more than 30 patients treated and outcome data to 1 year [28–36]

Study, year	PBA Cohort/total cohort	Study design	Primary Patency in PBA arm (%)	
			6 mths	12 mths
Trerotola; 2018 Trerotola; 2020	144/285	Prospective Multicentre RCT	63	36
Maleux; 2018	31/64	Prospective Multicentre RCT	65	39
Lookstein; 2020 Holden; 2022	160/330	Prospective Multicentre RCT	60	44
Karmota, 2020	30/60	Prospective Single centre RCT	90	67
Moreno-Sanchez; 2020	65/136	Prospective Multicentre RCT	58	47
Karunanithy, 2021	106/212	Prospective Multicentre RCT	85	59
Yin; 2021	83/161	Prospective Multicentre RCT	37	58

The overall reported target lesion primary patency at 6 and 12 months ranged between 37–90% and 36–67%, respectively. These data refer to the outcomes for PBA only reported within these DCB trials.

A systematic review and meta-analysis of studies comparing the outcomes between PBA and DCB reviewed a total of 11 studies [37]. Of these, there were 5 AVF only studies and 1 AVF subgroup [14, 32, 38–41]. The event rates (thrombosis, restenosis, etc.) at 6 months were 44.6% in the PBA group and 36.9% in the DCB group which were not significantly different. The difference in event rates at 12 months, primary patency at 6 and 12 months, as well as rate of complications, were also not significant. In summary, this paper demonstrated only modest improvement in primary patency with the use of DCB which was not statistically significant.

Other meta-analyses demonstrate improved outcomes for DCB compared to PBA. This is likely to be due to the selection of studies included in the analysis. Liao et al. only analysed RCTs, whereas Kennedy et al. included cohort and retrospective studies which have inherent selection bias [42, 43]. Additionally, these two meta-analyses excluded two RCTs which are likely to have affected the outcome of the results [38, 44].

#### Outcomes of PBA in Randomised Controlled Trials Comparing PBA with Stent Grafts

The major randomised controlled trials comparing the effectiveness of PBA with stent grafts are summarised in Table 4. All the studies report a significantly improved 6-month primary patency with the use of stent grafts compared to PBA [45–47]. Both the FLAIR [47] and RENOVA [46] studies showed restenosis occurred more frequently in the PBA group compared with the stent graft group. Of note, these two studies only included AVGs. The RESCUE [45] study included both AVG (46.2%) and AVF (53.8%). Additionally, the improvements were shown to have been sustained for up to 24 months in two of the studies [45, 46]. Although stent grafts have a greater cost, the study by Mohr et al. found that the reduction in the number of reinterventions seen in the stent graft group compensated for the increased initial cost of the stent grafts [48].

**Table 4:** Outcomes of plain balloon angioplasty in AVG stent graft trials with more than 30 patients treated [45–47]

Study, year	Angioplasty Cohort/total cohort	Study design	Lesion site	Primary Patency PBA (%)		Primary Patency Stent graft (%)	
				6 mths	12 mths	6 mths	12 mths
Haskal; 2010	93/190	Prospective Multicentre RCT	Venous anastomotic stenoses in AVG	23	NA	51	NA
Haskal; 2016	132/270	Prospective Multicentre RCT	Venous anastomotic stenoses in AVG	NA	25	NA	48
Falk, 2016	143/275	Prospective Multicentre RCT	In-stent restenosis in AVF and AVG	12.3	5.6	66.4	32.7

_*NA* not available_

### Summary of Outcomes Data for PBA

In summary, the primary patency (PP) following PBA in AVF at 6 and 12 months from cohort studies range between 42–63% and 23–50.5%. The PP for PBA in comparative studies against DCB at 6 and 12 months ranged between 37–90% and 36–67%, respectively. For AVGs, the PP at 6 and 12 months range between 27–61.3% and 10–41%. The PP for PBA in AVG stent graft trials at 6 and 12 months range from 12.3–23% and 5.6–25% [45–47].

## Discussion

There is significant heterogeneity in the published data on this subject. Many studies, especially the earlier ones, have small cohorts, mixed AVF and AVG populations which are not accurately defined, lesions that are not well described and poorly reported outcomes; these have not been included. Other studies have been excluded as they have a significant or undefined number of central occlusions and patients undergoing thrombectomy/thrombolysis for occlusions in addition to PBA, which is beyond the scope of this chapter. The overall evidence from the selected studies in this review supports the role of PBA as the mainstay of treatment of clinically significant stenosis in dialysis access. The outcomes are superior in AVF compared to AVG. Within the AVF cohort, the outcomes in forearm fistulas are better than the outcomes for upper arm fistulae. Although PBA provides good results that are comparable to surgical treatments, repeated angioplasties result in diminishing success rates with each procedure [5, 22]. This has implications on management strategy dependent on patient’s age, co-morbidities, and surgical options. Repeat angioplasty is still minimally invasive with lower complication rates compared to surgery and can be performed as a day case procedure. Surgical revision can thus be kept in reserve when angioplasty is no longer successful or is not meeting the most recent KDOQI treatment goal guidelines of no more than 3 interventions per year to maintain AV access before considering alternatives [5].

Cohort studies mostly demonstrate lower patency rates for AVG when compared to AVF [45–49]. This is thought to relate to the polytetrafluoroethylene (PTFE) grafts used in AVGs which have been shown to attract macrophages thought to result in more aggressive neo-intimal hyperplasia [50]. AVGs are also associated with more infections and higher morbidity and mortality [51, 52].

Although thrombosed fistulae were excluded from the main analysis, there is evidence to show that PBA has 80–100% success in restoring flow in occluded vessels [53, 54].

The outcomes for PBA in the DCB randomised controlled trials were generally better than those in the cohort studies (Table 3) except for the study by Yin et al. The poorer outcomes in the Yin paper may be related to the inclusion of access circuits with multiple stenoses with the choice of target lesion for the purpose of the study being left to the operator’s discretion [36]. The other papers excluded dialysis access circuits with more than one stenosis [29, 30] or only included those with one additional non target stenosis [33, 34]. Overall, it is possible that the strict inclusion and exclusion criteria of most of these studies explain the improved outcomes for PBA compared to that reported in cohort studies.

Conversely, the PBA outcomes in the AVG randomised controlled trials were worse when compared to those reported in the AVG cohort studies. Once again, this is likely to be due to patient selection. Of the three studies reviewed where 6-month primary patency was available, Haskal et al. treated patients with single stenoses at the venous anastomosis with the stent graft. Of note, 32% of the patients undergoing PBA in this study had axillary venous anastomosis which may have contributed to the poor outcome [47]. All patients in the Falk et al. study had in-stent restenosis, thus already likely to have poor outcomes compared to de novo lesions [45].

Stent grafts are used to overcome the problems arising from elastic recoil of a successfully treated lesion with PBA as well as preventing neo-intimal hyperplasia which can occur either if unstented or stented with a bare metal stent (BMS). The data from the FLAIR study show the use of stent grafts increase patency and durability of PBA; however, this was in a specific cohort of patients with venous anastomotic stenosis and additionally did not look at the longer-term outcome for these patients [47]. The high cost of stent grafts must be considered, and an overview must be taken over the lifetime of the patient and their dialysis access circuit. The use of DCB and stent grafts will be explored further in the relevant chapters.

The outcome of PBA is dependent on a variety of factors, which are inconsistently reported in various publications. The location of stenosis has not been specifically analysed. However, the response of lesions at various anatomical sites such as cephalic arch stenoses versus anastomotic lesions is likely to be different given the different haemodynamic forces. This is discussed at some length in a separate review [55]. The use of PBA in fistulas of different ages as well as primary treatment versus treatment of recurrent lesions may also affect the outcomes.

The limitations of this review is that it lacks a detailed analysis of some of these factors discussed which may influence outcome as well as study design, techniques and devices used. We have attempted to summarise the evidence available from cohort and comparative studies within the parameters defined in the materials and methods in order to provide a comprehensive and practical overview of the subject.

## Conclusion

Plain balloon angioplasty remains the first-line treatment for dialysis access stenosis but should be tailored to the individual patient’s needs. The outcomes are more favourable in AVFs compared to AVGs and for forearm fistulae compared with upper arm fistulae. The results also show that the greater the degree of stenosis of the treated lesion, the higher the rate of recurrence. Adjuncts to PBA such as the use of drug-coated balloons and stent grafts, as well as repeated angioplasty procedures can further prolong the life of dialysis access circuits, with surgical revision held in reserve when these options fail. Finally, it is important that PBA is only used in patients with both clinical and angiographic evidence of dysfunction and asymptomatic patients should not be treated.
